# Pharmacoepidemiology unpacked: a roadmap for junior researchers

**DOI:** 10.3389/fphar.2026.1801938

**Published:** 2026-06-25

**Authors:** Alyaa M. Ajabnoor

**Affiliations:** Department of Pharmacy Practice, Faculty of Pharmacy, King Abdulaziz University, Jeddah, Saudi Arabia

**Keywords:** observational research, pharmacoepidemiology, pharmacovigilance, real-world evidence, study design

## Abstract

Pharmacoepidemiology is an evolving discipline at the intersection of pharmacology, epidemiology, and clinical medicine, playing a pivotal role in evaluating real-world medication use, safety, and effectiveness. This review aims to provide early-career researchers with a structured and practical roadmap to navigate the field. The manuscript is organized around a step-by-step research framework, guiding readers from formulating a clear research question and protocol development, through study design selection and identification of appropriate data sources, to the operationalisation of key variables, including exposures, outcomes, and covariates. It further addresses statistical analysis and modelling, followed by critical evaluation of biases and limitations, and concludes with reporting and interpretation of findings. This integrated structure reflects the real-world progression of pharmacoepidemiologic research and supports coherent study planning and execution. Recent advancements in the field include the increasing use of real-world data, causal inference approaches, and advanced analytical methods, supported by large healthcare databases and evolving data infrastructures. In addition, the adoption of reporting standards such as STROBE and RECORD has improved transparency, reproducibility, and methodological rigor. This review provides an integrated, workflow-oriented perspective that bridges methodological concepts with practical application, equipping early-career researchers to design robust studies and contribute meaningfully to evidence-based drug safety and effectiveness research.

## 1 Introduction

Pharmacoepidemiology is defined as the study of the use of and the effects of drugs in large numbers of people, as described by Strom et al., (Eberhardt et al., 2021). Pharmacoepidemiology is a multidisciplinary field that intersects the principles of pharmacology, epidemiology, and clinical medicine, and focuses on the study of drug use and effects in large populations to inform clinical practice and public health policy (Eberhardt et al., 2021; Macfarlane, 1978; Upadhyay et al., 2019; Sabaté and Montané, 2023). The main purpose of pharmacoepidemiology is to deepen understanding of the benefits and risks associated with drug utilization, to support evidence-based decision-making in healthcare (Sabaté and Montané, 2023; Trevisan et al., 2021). Pharmacoepidemiology plays a critical role in post-marketing drug safety surveillance (pharmacovigilance), identifying adverse drug reactions (ADRs), rare or long-term side effects, and drug-drug interactions that may not be detected in pre-approval clinical trials (Sabaté and Montané, 2023; Trevisan et al., 2021; Basile et al., 2019). Post-marketing surveillance is essential for regulatory agencies and healthcare professionals to ensure ongoing patient safety. The field encompasses drug utilization studies, which analyze prescribing, dispensing, and consumption patterns. These studies help evaluate the quality of medication use, identify inappropriate prescribing, and assess the impact of interventions to improve drug use (Trevisan et al., 2021). Pharmacoepidemiology enables comparative effectiveness research, assessing the real-world performance of different drugs or treatment strategies in diverse patient populations, including those often excluded from clinical trials (e.g., elderly, pregnant women) (Sabaté and Montané, 2023; Trevisan et al., 2021; Takahashi et al., 2012). This research supports optimal therapeutic choices and personalized medicine. Findings from pharmacoepidemiologic studies inform health policy, guide regulatory decisions, and support cost-effectiveness analyses, ensuring that drug approval and reimbursement decisions are grounded in robust, real-world evidence (RWE) (Sabaté and Montané, 2023; Yoshida et al., 2022; Arlett et al., 2022). RWE, derived from real-world data (RWD) such as electronic health records, claims databases, and registries, is increasingly used to supplement clinical trial data for drug safety, effectiveness, and regulatory decisions (Sabaté and Montané, 2023; Yoshida et al., 2022; Arlett et al., 2022; Li et al., 2022). Regulatory agencies like the United States Food and Drug Administration (U.S.FDA) and European Medicines Agency (EMA) are actively incorporating RWE into their frameworks (Sabaté and Montané, 2023; Arlett et al., 2022). The expansion of large, diverse healthcare databases enables the study of rare events, long-term outcomes, and subgroups, enhancing the generalizability and impact of pharmacoepidemiologic research (Takahashi et al., 2012; Yoshida et al., 2022; Burden, 2019). Artificial intelligence (AI) and machine learning are transforming drug safety surveillance by automating ADR detection, analyzing unstructured data (e.g., clinical notes, social media), and enabling real-time pharmacovigilance. These technologies improve the efficiency and accuracy of safety monitoring but require high-quality data and transparent, explainable models (Sabaté and Montané, 2023; Burden, 2019; Dimitsaki et al., 2024; Shamim et al., 2024).

In recent years, pharmacoepidemiology has evolved into a multidisciplinary field at the intersection of pharmacology, epidemiology, and clinical medicine, driving advancements in drug safety, utilization, and regulatory decision-making. Despite the availability of comprehensive methodological resources, early-career researchers may face challenges navigating the multiple stages involved in designing and conducting pharmacoepidemiologic studies. Building on this need, this review aims to provide a structured, workflow-oriented overview of pharmacoepidemiology tailored for junior researchers and students. Rather than focusing on isolated methodological domains, the review integrates key components of the research process—including study design selection, data sources, variable operationalization, statistical approaches, bias assessment, reporting guidelines, and emerging methodological developments—within a unified framework aligned with the step-by-step progression of a pharmacoepidemiologic study. In addition, the review incorporates contemporary concepts such as real-world data frameworks, target trial emulation, and modern approaches to confounding control to better reflect current practice in the field.

## 2 Methods (literature search strategy)

This manuscript was conducted as a structured narrative review aimed at providing a workflow-oriented overview of pharmacoepidemiologic research for early-career researchers. A targeted literature search was performed using PubMed/MEDLINE and Google Scholar to identify relevant publications related to pharmacoepidemiology and real-world evidence. The search included articles published up to January 2026. Search terms included combinations of keywords such as “pharmacoepidemiology,” “pharmacovigilance,” “drug safety,” “real-world evidence,” “study design,” “data sources,” “bias,” “causal inference,” and “statistical methods,” combined using Boolean operators where appropriate. Eligible sources included methodological papers, narrative and systematic reviews, observational studies, reporting guidelines, textbooks, and regulatory or methodological guidance documents relevant to pharmacoepidemiologic research. Additional references were identified through manual screening of reference lists from key publications and guidance documents. Sources were selected based on their relevance to the scope and educational objectives of the review, with emphasis on foundational concepts, commonly applied methodologies, contemporary analytical approaches, and practical frameworks used in pharmacoepidemiology. Given the broad scope and heterogeneous nature of the literature, findings were synthesized narratively.

## 3 Pharmacovigilance and pharmacoepidemiology

Pharmacovigilance is a key component of pharmacoepidemiology, focusing specifically on the detection, assessment, understanding, and prevention of ADRs and other drug-related problems. Its primary tools include spontaneous reporting systems, post-marketing surveillance, literature reviews, observational studies, and advanced data mining techniques (Sharma, 2019; Shah Amran, 2021; Mishra et al., 2024; Basha and Sanghavi, 2022). These systems are crucial for identifying safety signals that may not emerge during pre-marketing clinical trials, as many ADRs only become apparent after widespread drug use (Shah Amran, 2021; Mishra et al., 2024). Pharmacoepidemiology has a broader scope, encompassing not only drug safety but also drug utilization, comparative effectiveness, and patterns of medication use in populations. It applies epidemiological methods to study both the beneficial and adverse effects of drugs and to evaluate exposures, outcomes, confounders, and effect modifiers. It often employs large healthcare databases and advanced statistical methods to address confounding and effect modification (Tang et al., 2025; Krishnan et al., 2024). The main types of pharmacoepidemiologic questions include drug safety, effectiveness, utilization patterns, and adherence/persistence that are summarized in Table 1.

**TABLE 1:** Applications of pharmacoepidemiology.

Application area	Core purpose	Typical approaches	Example application
Drug safety surveillance	Detection and evaluation of ADRs and safety signals in real-world settings	Spontaneous reporting systems, observational studies using RWD, signal detection methods, data mining and AI techniques	Adverse events associated with GLP-1 receptor agonists and SGLT2 inhibitors (Tang et al., 2025; Krishnan et al., 2024; Edwards et al., 2023)
Drug utilization research	Assessment of prescribing patterns, medication use, and healthcare delivery in populations	Analysis of EHRs, claims data, registries, and descriptive epidemiologic methods	National trends in statin prescribing (Kardas et al., 2024; Talic et al., 2022)
Comparative effectiveness research	Comparison of benefits and risks of alternative treatments in real-world settings	Cohort and case-control studies, propensity score methods, regression modeling, instrumental variable analysis	Comparative effectiveness of oral anticoagulants (Gatsonis et al., 2017; Concato et al., 2010)
Adherence and persistence research	Evaluation of medication-taking behavior and treatment continuation over time	Pharmacy refill data (MPR, PDC), self-reported measures, electronic monitoring, longitudinal modeling approaches	Adherence patterns across antihypertensive therapies (Pednekar et al., 2019; Schneider et al., 2019; Raebel et al., 2013; Malo et al., 2019; Schulz et al., 2016)

Abbreviations: ADRs, adverse drug reactions; AI, artificial intelligence; EHRs, electronic health records; GLP-1, glucagon-like peptide-1, receptor agonists; MPR, medication possession ratio; PDC, proportion of days covered; RWD, real-world data; SGLT2, sodium–glucose cotransporter two inhibitors.

## 4 Practical framework for conducting pharmacoepidemiologic studies

**FIGURE 1:**
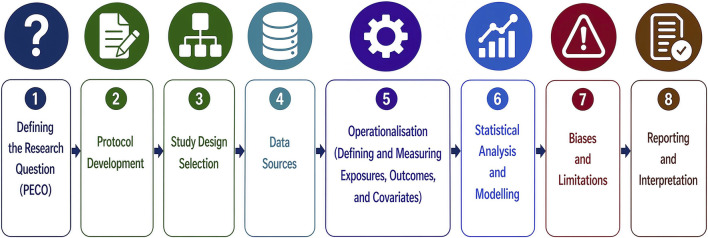
Conceptual workflow for conducting pharmacoepidemiologic studies. Abbreviations: PECO, Population, Exposure, Comparator.

Pharmacoepidemiologic research follows a structured and sequential process that ensures methodological rigor and validity of findings. As illustrated in Figure 1, this framework begins with the formulation of a clear and focused research question, and progresses through key steps including study design selection, identification of appropriate data sources, and careful definition of variables, exposures, and outcomes. Subsequent steps involve the application of suitable statistical methods and modelling strategies, alongside the identification and consideration of potential biases and limitations. The process concludes with the transparent reporting and interpretation of results. Although presented as a linear sequence, these steps are often iterative in practice, with decisions at each stage influencing and refining subsequent stages of the study.

### 4.1 Defining the research question

**FIGURE 2:**
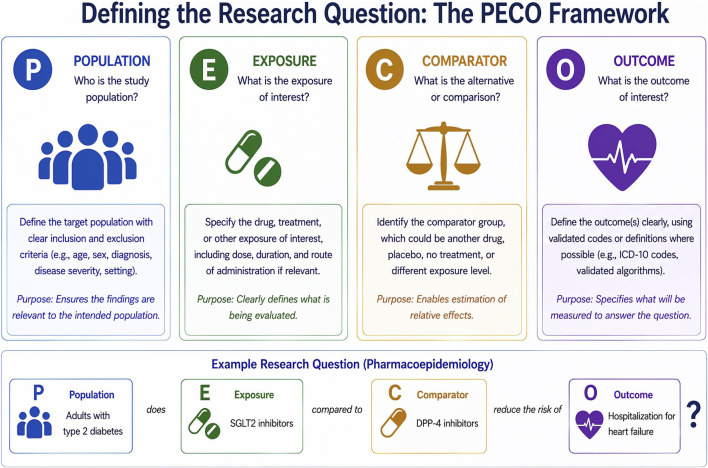
PECO-based framework for pharmacoepidemiologic research questions. Abbreviations: DPP-4, dipeptidyl peptidase-4 inhibitors; ICD-10, International Classification of Diseases, 10th Revision; PECO, population, exposure, comparator, outcome; SGLT2, sodium–glucose cotransporter two inhibitors.

A well-defined research question is a critical first step in pharmacoepidemiologic research, as it determines the choice of study design, data source, and analytical approach. The PICO framework—Population, Intervention (or exposure), Comparator, and Outcome—is commonly used to structure research questions in a clear and systematic manner (Figure 2), helping ensure that the research objective is focused, reproducible, and clinically relevant. However, while PICO is widely applied in clinical research, particularly randomized controlled trials, its direct application to observational pharmacoepidemiologic studies may be less appropriate. In these settings, treatments are not assigned but rather observed as exposures. Therefore, the PECO framework—Population, Exposure, Comparator, and Outcome—often provides a more accurate and intuitive structure for formulating research questions. PECO aligns more closely with the design and analytical approaches used in pharmacoepidemiology and is consistent with frameworks applied in other observational, exposure-based fields (Mintzker et al., 2023). As illustrated in Figure 2, each component of these frameworks contributes to defining a precise and answerable question. For example, a pharmacoepidemiologic question may be framed as: in adults with type 2 diabetes (Population), does exposure to SGLT2 inhibitors (Exposure) compared with DPP-4 inhibitors (Comparator) reduce the risk of hospitalization for heart failure (Outcome)? Clearly defining the research question at the outset facilitates appropriate methodological decisions and minimizes the risk of bias in subsequent stages of the study.

### 4.2 Protocol development

Protocols in pharmacoepidemiology are formal written plans that prespecify why and how a RWE study will be conducted. They are essential for ensuring methodological transparency, reproducibility, and validity of findings (ISPE, 2016; Langan et al., 2018). A well-developed protocol clarifies the research question, reduces data-driven decisions, and guides all stages of the study. According to the International Society for Pharmacoepidemiology (ISPE) Good Pharmacoepidemiology Practice (GPP), protocols should include key elements such as study objectives, design, population, exposure and outcome definitions, covariates, data sources, and timelines, as well as a prespecified statistical analysis plan addressing confounding and bias (ISPE, 2016; Langan et al., 2018; Wang et al., 2022). Protocol amendments should be documented and justified to maintain transparency (ISPE, 2016). Modern protocols also emphasize explicit definition of the causal question or estimand—i.e., the precise effect of an exposure in a specified population under defined conditions, including handling of intercurrent events (Luijken et al., 2023). In addition, detailed documentation of data sources, measurement strategies, and bias mitigation approaches is required. Standardized templates such as the HARmonized Protocol Template to Enhance Reproducibility (HARPER), developed by ISPE and the International Society for Pharmacoeconomics and Outcomes Research (ISPOR), and methodological guidance from the European Network of Centres for Pharmacoepidemiology and Pharmacovigilance (ENCePP), support consistent and transparent protocol development (ISPE, 2016; Wang et al., 2022). This is particularly important in multi-database studies requiring harmonized definitions and analyses. Protocol registration in public repositories (e.g., EU PAS Register, ENCePP, ClinicalTrials.gov) is encouraged to enhance transparency and reduce selective reporting (ISPE, 2016). Alignment with reporting guidelines such as RECORD-PE and STROBE further ensures clear and reproducible communication of study methods and findings (Langan et al., 2018; Benchimol et al., 2015; Elm et al., 2007). Table 2 provides an overview of the main elements to be considered when developing a pharmacoepidemiologic study protocol, including key methodological components, analytical considerations, and aspects related to transparency and reporting.

**TABLE 2:** Key components of a pharmacoepidemiologic study protocol.

Component	Key elements	Purpose
Title and identifiers	Study title, version, date, registry number	Ensures traceability and transparency
Roles and funding	Investigators, institutions, sponsors	Clarifies accountability and potential conflicts
Objectives and rationale	Study aims, clinical and scientific justification	Defines research purpose
Study design and methods	Design, population, exposure, outcomes, covariates, timelines	Guides study implementation
Statistical analysis plan	Models, confounding control, bias handling	Ensures analytical transparency
Data and measurement	Data sources, coding systems, variable construction	Supports reproducibility
Bias and confounding strategy	Design and analytical approaches	Improve internal validity
Transparency and registration	Protocol registration, reporting standards (RECORD-PE/STROBE)	Enhances credibility and reproducibility
Amendments	Documented changes with justification	Maintains transparency over time

Abbreviations: RECORD-PE, reporting of studies conducted using observational routinely collected health data for pharmacoepidemiology; STROBE, strengthening the reporting of observational studies in epidemiology.

### 4.3 Study design selection

**FIGURE 3:**
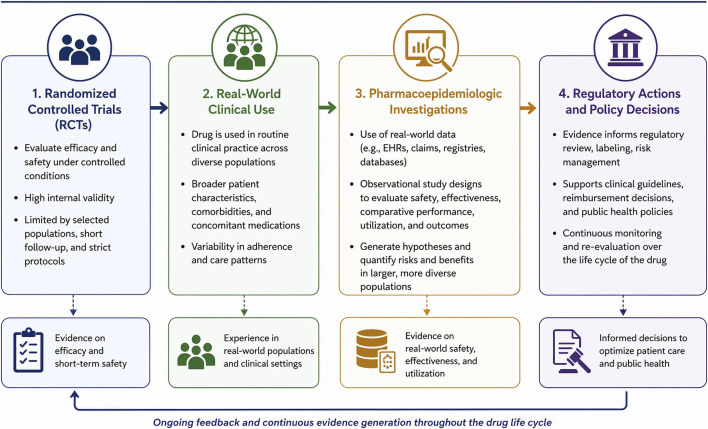
Sequence of drug evidence from randomized controlled trials to real-world pharmacoepidemiologic research and regulatory decision-making. Abbreviations: EHRs, Electronic Health Records; RCTs, Randomized Controlled Trials.

While randomized controlled trials (RCTs) remain the gold standard for establishing causal relationships between interventions and outcomes, their strict protocols, limited sample sizes, and controlled settings often restrict their generalizability to real-world populations. In contrast, pharmacoepidemiology relies heavily on observational studies, which leverage real-world data to assess the safety, effectiveness, and utilization of medications in diverse clinical settings. Figure 3 illustrates the progression of evidence generation, beginning with RCTs, followed by real-world clinical use and pharmacoepidemiologic investigations, and ultimately informing regulatory actions and policy decisions.

**FIGURE 4:**
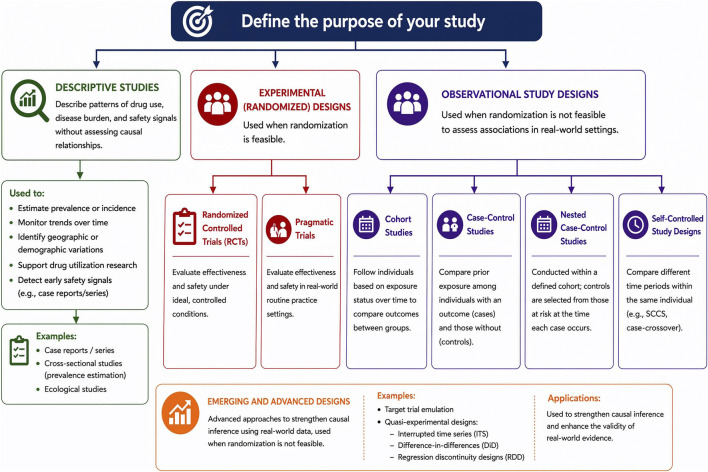
Decision framework for selecting appropriate study designs in pharmacoepidemiology. Abbreviations: DID, Difference-in-Differences; ITS, Interrupted Time Series; RCTs, Randomized Controlled Trials; RDD, Regression Discontinuity Design; SCCS, Self-Controlled Case Series.

Selecting an appropriate study design is a critical first step in pharmacoepidemiologic research, as it determines the validity, reliability, and interpretability of findings. The choice depends on the research question, the nature of drug exposure, the availability of data, and whether randomization is feasible or ethical as illustrated in Figure 4. Importantly, the trajectory of evidence generation is not always strictly linear, as RCTs may be not feasible or unethical in certain contexts—such as when evaluating harmful exposures, rare outcomes, or long-term effects—necessitating reliance on observational or real-world data (Franklin et al., 2022). Broadly, study designs can be divided into experimental designs—such as RCTs and observational designs, which are either descriptive (e.g., drug utilization studies) or analytical (e.g., cohort, case-control, and self-controlled designs). Based on this, the following section explores the major study designs in pharmacoepidemiology, highlighting their structure, strengths, limitations, and typical applications.

#### 4.3.1 Descriptive study designs

**FIGURE 5:**
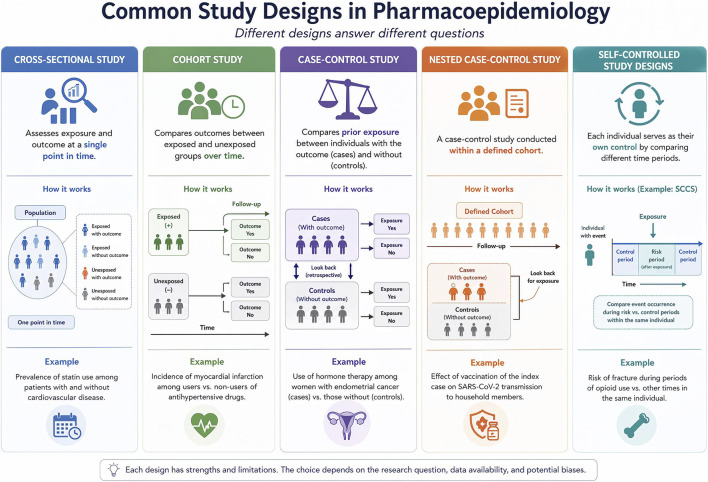
Schematic representation of key observational study designs in pharmacoepidemiology. Abbreviations: SCCS, Self-Controlled Case Series.

Descriptive pharmacoepidemiologic studies map how drugs are used and what happens in routine care, without formal comparison groups. They underpin rational prescribing, early safety signal detection, and understanding of population-level patterns that later analytic studies investigate more deeply (Rasmussen et al., 2022). Descriptive studies are often easy, quick, and inexpensive to conduct, as they typically use existing data or require only a single data collection point (Figure 5). They are valuable for identifying new or rare adverse drug reactions and generating hypotheses for further analytic research (Sabaté and Montané, 2023). Useful for estimating disease or drug use burden, monitoring trends over time, and identifying geographic or demographic variations (Aggarwal and Ranganathan, 2019; Noe and Gelfand, 2018). Their findings can inform healthcare resource allocation and public health planning. Descriptive studies are used for drug utilization research to describe how medications are prescribed and used in populations, such as tracking the use of anticancer drugs in national health databases. It can be used for early detection of unexpected or rare adverse drug reactions, often through case reports or case series. As it allows for the assessment of prevalence or incidence of drug-related events to guide policy and intervention (Aggarwal and Ranganathan, 2019; Noe and Gelfand, 2018). However, descriptive studies cannot establish causality, as they lack comparison groups and do not test hypotheses. Prone to confounding, selection bias, and measurement errors, especially when using secondary or administrative data. Case reports and series may reflect chance occurrences and may not be generalizable. Findings often require confirmation through analytic studies (e.g., cohort or case-control) to determine true associations (Aggarwal and Ranganathan, 2019; Noe and Gelfand, 2018). Example of descriptive studies; case report/series such as initial identification of rare side effects to a new drug through a case report and subsequent case series of similar reports on the same side effect. Cross-sectional study such as surveying the prevalence of statin use in a population at a single point in time. Ecological study such as examining the relationship between regional antibiotic sales and rates of antibiotic resistance.

#### 4.3.2 Experimental study designs

Experimental studies involve the active allocation of an intervention by the researcher, typically through randomization, to evaluate its effect on health outcomes. These designs are primarily represented by RCTs and pragmatic trials, as illustrated in Figure 4. Randomization ensures that study participants are assigned to intervention or comparator groups by chance, which minimizes confounding and selection bias, thereby providing the strongest level of evidence for causal inference.

##### 4.3.2.1 Randomized controlled trials

Randomized controlled trials are considered the gold standard for evaluating the efficacy and safety of medical interventions under controlled conditions. Participants are randomly assigned to receive either the intervention or a comparator (e.g., placebo or standard treatment), and outcomes are assessed prospectively. RCTs provide high internal validity due to randomization and controlled study conditions, which minimize bias and confounding (Monti et al., 2018). They allow for clear causal inference and are widely used to evaluate drug efficacy, safety, and dose-response relationships, particularly in pre-marketing settings. Standardized protocols, strict eligibility criteria, and structured follow-up enhance the precision and reliability of outcome assessment. Despite their strengths, RCTs often have limited generalizability, as they typically include selected patient populations and operate under controlled conditions that may not reflect routine clinical practice (Deaton and Cartwright, 2018). In pharmacoepidemiology, their use is often constrained by ethical, logistical, and financial considerations, especially when studying rare adverse events, long-term outcomes, or vulnerable populations.

##### 4.3.2.2 Pragmatic trials

Pragmatic trials are a type of randomized study designed to evaluate the effectiveness of interventions in real-world clinical practice settings, rather than under ideal experimental conditions. Pragmatic trials enhance external validity by reflecting routine healthcare practice (Staa et al., 2012). They typically include broader and more diverse patient populations, flexible treatment protocols, and usual care comparators. These features make them particularly useful for generating RWE to inform clinical decision-making, health policy, and guideline development. They often utilize routine healthcare data sources, improving feasibility and scalability. Compared to traditional RCTs, pragmatic trials may have reduced control over confounding factors, adherence, and intervention fidelity, which can affect internal validity. Variability in clinical practice settings and data quality may introduce measurement bias and complicate interpretation of results (Mentz et al., 2016).

#### 4.3.3 Analytical study designs

##### 4.3.3.1 Cohort studies

A cohort in pharmacoepidemiology refers to a group of individuals who share common exposure or characteristics such as starting a specific medication—within a defined time period (Figure 5). These individuals are followed over time to observe and compare the occurrence of outcomes (e.g., adverse events, effectiveness) between those exposed to a drug and those who are not, or between different exposure groups (such as users of two different drugs) (Creagh, 1992; Pottegård, 2022). Cohorts are often defined by drug initiation but can also include non-users or previous users as comparators. Individuals are tracked from a defined entry point (e.g., date of first prescription) until the outcome occurs, they leave the study, or the study ends. The rate or risk of outcomes is compared between exposure groups to estimate increased, decreased, or unchanged risk associated with the drug. Cohort studies can assess several outcomes from a single exposure, such as both effectiveness and adverse events (Creagh, 1992; Pottegård, 2022). Cohort studies allow calculation of incidence rates and relative risks, providing clear measures of association between drug exposure and outcomes. Temporal clarity as it ensures that exposure precedes outcome, supporting causal inference. Additionally, it reflects actual medication use and patient populations outside of controlled trial settings, making the findings more generalizable (Creagh, 1992; Pottegård, 2022). However, differences between exposure groups (e.g., health status, indication for drug use) can introduce confounding, which must be addressed through design or analysis (e.g., matching, regression, propensity scores). Patient characteristics and prescribing practices can affect group comparability, especially when guidelines influence treatment choices which could introduce selection bias (Creagh, 1992; Pottegård, 2022). During the early COVID-19 pandemic, cohort designs were used to assess medication-related risks. For example, an international study used electronic health records to compare outcomes among users of renin–angiotensin system blockers *versus* other antihypertensives, evaluating COVID-19 diagnosis and related hospitalizations (Morales et al., 2021).

##### 4.3.3.2 Case-control studies

A case-control study identifies individuals with a specific outcome or disease (cases) and compares their prior exposure to a drug or other factor with that of individuals without the outcome (controls). The proportion of exposure among cases and controls is compared to estimate the association between the drug and the outcome (Pottegård, 2022; Stolley, 1992). Case-control studies are Ideal for investigating suspected associations between drugs and rare or delayed adverse events. They are also useful when exposure data are expensive or difficult to obtain for an entire population and are often used to generate hypotheses for further study in larger or prospective designs (Pottegård, 2022; Stolley, 1992). Case-control studies are particularly useful for studying rare diseases or adverse drug reactions, as it does not require following large populations over time. Exposure is assessed retrospectively, after the outcome has occurred, making the design relatively quick and cost-effective (Figure 5). Controls should represent the background exposure in the population from which cases arise, ensuring comparability. The main measure of association is the odds ratio, which estimates the relative odds of exposure among cases *versus* controls (Pottegård, 2022; Stolley, 1992). Careful selection of controls is critical; poor selection can introduce selection bias. Reliance on accurate exposure data (e.g., prescription records or patient recall) can affect validity and introduce recall/information bias. Unlike cohort studies, case-control studies do not provide direct incidence rates or relative risks as it cannot directly estimate incidence (Pottegård, 2022; Stolley, 1992). The most cited example of a case–control study is the Doll and Hill investigation of cigarette smoking and lung cancer (Doll and Hill, 1950), alongside other early cancer studies (pipe smoking and lip/oral cancer; reproductive factors and breast cancer) (Breslow, 2024; Breslow, 1996). These studies showcased how the case–control design can uncover strong risk–disease relationships, especially for rare outcomes, and still serve as teaching models today.

##### 4.3.3.3 Nested case-control studies

A nested case-control study is conducted within a well-defined cohort (Figure 5). As cases (individuals who develop the outcome of interest) arise, a sample of controls is selected from cohort members who have not yet developed the outcome at the time each case occurs. This design allows for matching on factors like age, calendar time, and disease duration, and enables detailed exposure assessment only for the selected cases and controls (Sabaté and Montané, 2023; Pottegård, 2022). Nnested case-control studies are considered efficient as its only use a subset of the cohort (cases and matched controls). The controls are selected from those at risk at the time of each case, preserving the temporal relationship between drug exposure and outcome. Matching and risk-set sampling allow for control of confounding variables and better quantification of time-dependent exposures (Sabaté and Montané, 2023; Pottegård, 2022). Also, selection bias is minimized since both cases and controls come from the same cohort, and information bias is reduced because exposure data are often collected before the outcome occurs. Nested case-control studies are widely used to study rare adverse drug reactions or outcomes where full-cohort analysis would be costly or impractical. Its well-suited for evaluating the effects of drugs with changing exposure status over time, using advanced statistical methods like marginal structural models. It is particularly advantageous when biological samples or detailed data are only available for a subset of the cohort (Sabaté and Montané, 2023; Pottegård, 2022). Careful selection of controls is critical; poor selection can introduce selection bias. Reliance on accurate exposure data (e.g., prescription records or patient recall) can affect validity and introduce recall/information bias. Unlike cohort studies, case-control studies do not provide direct incidence rates or relative risks estimates (Sabaté and Montané, 2023; Pottegård, 2022). Nested case-control designs have been used to efficiently evaluate transmission dynamics in real-world settings. For example, a study in England assessed the impact of COVID-19 vaccination on household transmission by selecting cases (secondary infections within households) and controls (non-infected household members) from a defined cohort of confirmed COVID-19 cases. Exposure was defined by the vaccination status of the index case, and the analysis estimated odds ratios for transmission risk, demonstrating reduced transmission from vaccinated individuals (Harris et al., 2021).

##### 4.3.3.4 Self-controlled study designs

Such as the self-controlled case series (SCCS) and case-crossover (CCO) designs, are increasingly used in pharmacoepidemiology to evaluate associations between drug exposures and outcomes by comparing different time periods within the same individual (Figure 5) (Cadarette et al., 2021; Iwagami and Takeuchi, 2021; Bots et al., 2025). By using each individual as their own control, these designs inherently adjust for all confounders that do not change over time (e.g., genetics, chronic health status, lifestyle), even if they are unmeasured or unknown (Iwagami and Takeuchi, 2021; Bots et al., 2025). These studies do not require a separate control group, simplifying design and data collection. Resources saved by not extracting data for non-cases can be used to include more cases, increasing statistical power. They are particularly advantageous when using large healthcare databases where some confounders are not recorded. These types of study designs are suitable for studying acute adverse drug reactions, vaccine safety, and drug-drug interactions, especially when exposures are transient and outcomes are abrupt. They are also useful for initial signal detection in pharmacovigilance. Moreover, they are ideal when time-invariant confounders are not available in the data source (Iwagami and Takeuchi, 2021; Bots et al., 2025). They are best suited for transient exposures and abrupt outcomes, not appropriate for long-term exposures or insidious outcomes. It cannot control for confounders that change over time (e.g., age, concomitant medications, disease progression). If exposure trends change during the study period, results may be biased. Each self-controlled design (SCCS, CCO) has unique assumptions that must be met; violation of these can lead to biased estimates. Finally, these study designs are inappropriate for outcomes that affect subsequent exposure or for exposures that are not intermittent (Iwagami and Takeuchi, 2021; Bots et al., 2025). Example of SCCS, a study using the Clinical Practice Research Datalink (CPRD) assessed the risk of fractures following opioid use by comparing periods of exposure and non-exposure within individuals, estimating the relative incidence across different risk windows (Peach et al., 2021). An example for case-crossover study: a nationwide case-crossover study evaluated the short-term risk of stroke associated with nonsteroidal anti-inflammatory drug (NSAID) use by comparing each patient’s exposure during the period immediately before the stroke (case period) with earlier control periods within the same individual. Using healthcare claims data, the study found an increased risk of both ischemic and hemorrhagic stroke during periods of NSAID exposure, illustrating the utility of this design for assessing transient drug effects on acute outcomes (Chang et al., 2010).

### 4.4 Data sources in pharmacoepidemiology

Pharmacoepidemiology relies on a variety of data sources to study drug use, safety, and effectiveness in real-world settings. These data sources can be broadly classified based on the type and origin of data, reflecting how information is generated and collected within healthcare and research systems. The main data sources include administrative claims databases, EHRs, disease and drug registries, pharmacy dispensing databases, and patient-generated health data. Each source has unique strengths and limitations, and the choice depends on the research question and required data elements. Table 3 summarizes commonly used data sources in pharmacoepidemiology, highlighting their key features and examples.

**TABLE 3:** Summary of data source types in pharmacoepidemiologic research and their examples.

Data source type	Key features	Examples
Administrative claims databases	Large, routinely updated, including insurance claims, diagnoses, procedures, and prescriptions	HIRD (US) (Barron et al., 2025), Audifarma (Colombia) (Franco and Vizcaya, 2020), Danish national database of reimbursed prescriptions (Johannesdottir et al., 2012)
Electronic health records (EHRs)	Detailed clinical data from healthcare providers; include diagnoses, lab results, and prescriptions	United Kingdom Clinical Practice Research Datalink (CPRD) (García Rodríguez and Pérez Gutthann, 1998), hospital-based EMR systems (Yoshida et al., 2022; Franco and Vizcaya, 2020)
Disease/drug registries	Organized systems collecting uniform data on specific diseases or drugs; useful for rare conditions	Cancer registries, product registries (Yoshida et al., 2022; Franco and Vizcaya, 2020; Johannesdottir et al., 2012)
Pharmacy dispensing databases	Capture data on drugs dispensed at pharmacies; useful for drug utilization studies	MarketScan (US) (Alatorre et al., 2017)
Patient generated health databases	Data from wearables, mobile devices, or patient surveys; increasingly used for real-world evidence (Bourke et al., 2020)	
Government/population databases	National health datasets, often linkable to other sources	The Nordic national health registers (Prami et al., 2021)

Abbreviations: CPRD, clinical practice research datalink; EHRs, electronic health records; EMR, electronic medical records; HIRD, health insurance review and assessment service database; US, united states.

The data source must contain the necessary information (e.g., exposures, outcomes, confounders) to address the specific research question and should meet key quality criteria, including reliability, completeness, and accuracy. Combining multiple sources can enhance data depth and support complex studies. Frequency of updates and ease of access vary across sources and countries. There are wide range of data sources underpin pharmacoepidemiologic research, each with distinct advantages and limitations. Careful selection and understanding of these sources are essential for generating robust, real-world evidence.

### 4.5 Operationalization

This section focuses on the operationalization of variables in pharmacoepidemiologic research, encompassing the definition and measurement of exposures, outcomes, and covariates (including confounders). Building on the selected data sources, clinical and research concepts are translated into measurable variables through appropriate definitions, coding strategies, and analytical considerations. Particular attention is given to issues such as dynamic exposure patterns, outcome validity, confounder identification using causal frameworks, and potential measurement error. These decisions are critical, as they directly influence the validity, interpretability, and overall quality of study findings.

#### 4.5.1 Exposure definitions, and measurement

Exposure can be defined in several ways, including current use, cumulative dose, duration, and time-varying patterns, and these definitions should be selected to reflect the underlying research question and real-world patterns of medication use. Importantly, drug exposure is not a single fixed construct, but rather a multidimensional concept describing how, when, and for how long medications are used in a population.

In real-world settings, drug use is often dynamic, as patients may initiate, discontinue, switch therapies, or use medications concurrently. As highlighted in methodological literature, individual-level dispensing data allows researchers to characterize not only exposure status but also patterns of use such as persistence (continuation of therapy), implementation (extent to which dosing follows the prescribed regimen), switching, and concomitant drug use (Rasmussen et al., 2022). To account for these complexities, methodological approaches such as treatment episode construction, time-varying covariate modelling, and weighted cumulative exposure models are commonly applied (Table 4) (Pazzagli et al., 2018). These approaches are particularly important when studying longitudinal drug use and time-dependent effects.

**TABLE 4:** Key dimensions of drug exposure definition and associated methodological considerations.

Exposure dimension	Description/Use	Key challenge
Timing (e.g., current use)	Exposure status at a defined point or risk window	May not capture past or cumulative effects
Cumulative dose	Total drug exposure over a specified period	Requires accurate dose and duration data
Duration of use	Length of continuous exposure (treatment episodes)	Defining start/stop dates and gaps
Time-varying exposure	Captures changes in exposure over time	Complex modelling and time-dependent confounding
Adherence/stockpiling	Measures continuity of use (e.g., proportion of days covered)	Misclassification due to incomplete or indirect measures

Exposure is typically derived from prescription or dispensing records, which represent proxies for actual medication intake rather than direct measures of use. As a result, assumptions regarding treatment start and stop dates, adherence, and handling of gaps or stockpiling are required. In addition, distinguishing between treatment switching and concomitant use can be challenging when relying on dispensing data, and may introduce misclassification if not carefully addressed (Rasmussen et al., 2022). Standardized metrics such as the Defined Daily Dose (DDD) are frequently used to facilitate comparisons across populations; however, they may not reflect individual-level dosing, clinical indications, or treatment intensity, and should therefore be interpreted with caution (Pazzagli et al., 2018). Importantly, different exposure definitions may yield different effect estimates, underscoring the need for transparent reporting and, where appropriate, sensitivity analyses using alternative exposure definitions.

Methodological considerations - detailed reporting of exposure definitions—including risk windows, induction periods, and handling of switching or adherences essential for study reproducibility and interpretation (Hempenius et al., 2020). For complex, longitudinal exposure patterns, advanced statistical models (e.g., marginal structural models, weighted cumulative exposure models) are recommended to minimize bias and accurately estimate effects (Pazzagli et al., 2018; Wood et al., 2022; Danieli et al., 2020; Kelly et al., 2024). Exposure assessment should be tailored to the drug, disease, and research question, especially for long-term or intermittent therapies (Wood et al., 2022).

#### 4.5.2 Outcome types, measurements, and challenges

Outcomes are central to pharmacoepidemiology, as they determine the effects—both beneficial and harmful—of drug exposures in real-world populations. Clinical outcomes, adverse events, healthcare utilization, and patient-reported measures are commonly studied, each with specific considerations for measurement and validity.

*Type of outcomes* – (Eberhardt et al., 2021) the most frequently used are clinical outcomes including disease diagnoses, hospitalizations, and mortality. For example, 96% of recent Danish pharmacoepidemiology studies used clinical outcomes, with diagnosis (66%) and mortality (38%) being the most common subcategories (Thor Petersen et al., 2022). (Macfarlane, 1978) Adverse events such as detection of drug-related side effects or complications (e.g., gastrointestinal toxicity or suicidal behavior) are a key focus. Accurate classification and validation of these outcomes are critical, as misclassification can bias results (Weinstein et al., 2023). (Upadhyay et al., 2019) Healthcare utilization and costs like healthcare visits, medication adherence, and costs are also assessed, though less frequently than clinical endpoints (Thor Petersen et al., 2022). (Sabaté and Montané, 2023) Patient-reported outcomes such as quality of life or symptom scores are used but remain relatively rare in large database studies (Thor Petersen et al., 2022).

*Measurements* - Most outcomes are identified using diagnostic codes, procedure codes, or mortality records from national registries or administrative databases (Thor Petersen et al., 2022; Weinstein et al., 2023). The accuracy and validity of outcome definitions (e.g., using ICD codes) are essential. Validation studies and algorithm refinement are recommended to minimize misclassification and improve reliability. Key challenges in outcome detection are misclassification, incomplete data, and lack of sensitivity in coding that can lead to underestimation or bias in associations between drug exposure and outcomes (Weinstein et al., 2023).

#### 4.5.3 Measurement of covariates

**FIGURE 6:**
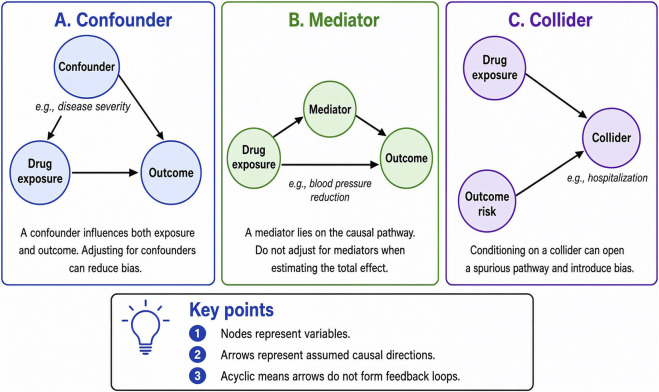
Directed acyclic graphs (DAGs) illustrating confounders, mediators, and colliders in pharmacoepidemiology. **(A)** Confounder. **(B)** Mediator. **(C)** Collider.

Covariates in pharmacoepidemiologic studies include demographic, clinical, and healthcare-related variables that may influence both drug exposure and outcomes. Accurate identification and measurement of these variables are essential to control for confounding and ensure valid estimation of treatment effects. Confounding refers to the distortion of the association between exposure and outcome by extraneous variables, and both measured and unmeasured confounding remain central challenges in studies using routinely collected data (Prada-Ramallal et al., 2019). Careful selection of covariates is therefore critical. Current methodological guidance recommends the use of subject-matter knowledge alongside causal frameworks, such as directed acyclic graphs (DAGs) (Figure 6), to identify true confounders and avoid inappropriate adjustment for mediators, colliders, or instrumental variables, which may introduce bias (Tennant et al., 2021). Traditional approaches to confounder selection, such as change-in-estimate criteria, may be unreliable when exposure or covariates are measured with error. Evidence suggests that data-driven selection alone is insufficient, and that thoughtful consideration of the research question, data structure, and measurement quality is required when defining covariates (Lee and Burstyn, 2016).

A major challenge in pharmacoepidemiologic research is that covariates are often measured with error or misclassification, particularly when derived from administrative claims or electronic health records. Such measurement error can lead to biased and imprecise estimates of exposure–outcome associations. Validation studies are therefore essential to assess the accuracy of coding algorithms used to define exposures, outcomes, and covariates. In addition, sensitivity analyses and validation approaches can be used to evaluate the robustness of findings in the presence of measurement error (Keogh et al., 2020; Brakenhoff et al., 2018).

Finally, the timing of covariate measurement must be carefully aligned with the exposure and outcome periods. Covariates are often measured at baseline; however, in longitudinal studies, many covariates vary over time and may require time-varying modelling approaches. Failure to appropriately account for time-dependent confounding may lead to biased estimates, particularly in studies of long-term or dynamic treatment patterns.

### 4.6 Statistical analysis and modelling

Pharmacoepidemiology employs a range of statistical methods to evaluate drug safety, effectiveness, and utilization in real-world populations. These methods can be broadly classified according to their primary analytical purpose, including estimating associations, controlling for confounding, analyzing time-to-event data, and assessing robustness to bias. Increasingly, advanced approaches—such as high-dimensional propensity scores, target trial emulation, and machine learning methods—are used to enhance causal inference and improve control of confounding in complex real-world data.

#### 4.6.1 Estimating associations

Regression-based models are widely used to quantify the relationship between drug exposure and outcomes. Common approaches include logistic regression (for binary outcomes), Poisson regression (for rates), and Cox proportional hazards models (for time-to-event data) (Takahashi et al., 2012; Macedo et al., 2020; Rippin et al., 2024). These models allow adjustment for multiple covariates and can accommodate both fixed and time-varying exposures (Rippin et al., 2024; Laura and Xiaojuan, 2021).

#### 4.6.2 Controlling for confounding

Controlling for confounding is central to pharmacoepidemiologic analysis. In addition to multivariable regression, propensity score (PS) methods—including matching, stratification, weighting, and covariate adjustment—are widely used to balance observed confounders between treatment groups (Takahashi et al., 2012; Arbogast and Ray, 2009; Uddin et al., 2016).

##### 4.6.2.1 Propensity scores

More advanced approaches include high-dimensional propensity scores (hdPS), which use automated algorithms to select large numbers of covariates from healthcare databases, improving control of confounding in high-dimensional data settings. Disease risk scores may also be used as an alternative or complement to propensity scores. Compared with traditional investigator-specified models, hdPS has been shown to improve confounding control and move estimates closer to those observed in randomized trials (Arbogast and Ray, 2009).

##### 4.6.2.2 Machine learning

Recent developments incorporate machine learning methods—such as Super Learner, Least Absolute Shrinkage and Selection Operator (LASSO), and random forests—to enhance covariate selection and propensity score estimation. These approaches can improve covariate balance, reduce bias, and increase precision, although concerns regarding overfitting and interpretability require careful consideration (Karim, 2025; Karim et al., 2018).

##### 4.6.2.3 Target trial emulation

In addition, target trial emulation has emerged as a structured framework for designing observational studies to mimic randomized controlled trials. By explicitly defining eligibility criteria, treatment strategies, time zero, and follow-up, target trial emulation helps reduce biases such as immortal time bias and prevalent user bias, thereby improving causal interpretation (Hernán et al., 2022).

#### 4.6.3 Time-to-event analysis

Time-to-event (survival) analysis is essential in pharmacoepidemiology, as many outcomes occur over time. Methods such as Cox proportional hazards models account for censoring and varying follow-up durations and can incorporate time-dependent exposures and covariates (Rippin et al., 2024; Laura and Xiaojuan, 2021).

#### 4.6.4 Addressing unmeasured confounding and bias

Residual confounding and bias remain important concerns. Methods such as instrumental variable (IV) analysis and self-controlled designs (e.g., case-crossover and self-controlled case series) can help address unmeasured confounding (Laura and Xiaojuan, 2021; Uddin et al., 2016; Ertefaie et al., 2017). In addition, negative control outcomes and exposures are increasingly used to detect residual bias, particularly unmeasured confounding. These approaches are primarily used for bias detection but may also support bias adjustment and calibration in some settings (Uddin et al., 2016; Dhopeshwarkar et al., 2024). Recent methodological work has demonstrated that combining negative control outcomes and exposures can improve adjustment for biases such as healthy user effects, enhancing causal interpretation (Levintow et al., 2023; Li et al., 2025). Broader methodological frameworks also emphasize complementary approaches, including quantitative bias analysis and sensitivity analyses, to assess the potential impact of unmeasured confounding (Uddin et al., 2016).

#### 4.6.5 Machine learning and data-driven approaches

Machine learning methods are increasingly applied in pharmacoepidemiology for confounder selection, risk prediction, and pattern recognition in large healthcare datasets. These approaches can complement traditional statistical models by capturing complex relationships and improving variable selection, although their use requires careful consideration of causal assumptions and interpretability (Karim, 2025; Schneeweiss et al., 2017; Rassen et al., 2023).

#### 4.6.6 Sensitivity analyses

Sensitivity analyses are essential to evaluate the robustness of study findings to different assumptions and potential biases. These may include testing alternative exposure definitions, varying model specifications, and assessing the impact of unmeasured confounding. Such analyses enhance transparency and strengthen the credibility of results (Brown et al., 2024; Fu et al., 2021).

#### 4.6.7 Causal inference in pharmacoepidemiology

Causal inference is central to pharmacoepidemiology, as studies often aim to estimate the causal effects of drug exposures using observational data. Unlike traditional association-based approaches, causal frameworks explicitly address confounding, selection bias, and time-dependent processes. DAGs are used to represent assumed relationships between variables and to guide appropriate confounder selection (Tennant et al., 2021). Target trial emulation provides a structured approach to designing observational studies that mimic randomized trials, improving causal interpretation (Hernán et al., 2022). For longitudinal settings with time-varying exposures, marginal structural models (MSMs) (Robins et al., 2000) and other g-methods—including inverse probability weighting, the parametric g-formula, and g-estimation—are used to appropriately adjust for time-varying confounding affected by prior treatment (Mansournia et al., 2017), a common challenge in pharmacoepidemiology. Together, these approaches form the foundation of modern causal inference in pharmacoepidemiology and support more robust evaluation of drug safety and effectiveness.

Causal inference is central to pharmacoepidemiology because many studies aim to estimate the effect of a medication on a clinical outcome using observational data rather than randomized treatment allocation. In this setting, the main methodological challenge is that treatment decisions are influenced by patient characteristics, disease severity, comorbidities, contraindications, physician preference, healthcare access, and previous treatment history. Therefore, observed differences between exposed and unexposed patients may reflect underlying differences between patients rather than the causal effect of the drug itself. Modern causal inference approaches help researchers define the causal question more explicitly, identify potential sources of bias, and select design and analytical strategies that approximate the conditions of a randomized trial.

Directed acyclic graphs (DAGs) are useful tools for making causal assumptions transparent. They visually represent the assumed relationships among exposure, outcome, confounders, mediators, colliders, and selection mechanisms. In pharmacoepidemiology, DAGs can help distinguish variables that should be adjusted for, such as common causes of treatment and outcome, from variables that should not be adjusted for, such as mediators on the causal pathway or colliders that may introduce bias. To improve the practical clarity of this concept for novice researchers, Figure 6 illustrates three common DAG structures—confounder, mediator, and collider—and summarizes how each structure affects adjustment decisions. Therefore, DAGs support more transparent confounder selection and reduce the risk of inappropriate adjustment (Tennant et al., 2021).

Target trial emulation provides a structured framework for designing observational pharmacoepidemiologic studies by explicitly specifying the protocol of the hypothetical randomized trial that the observational study attempts to emulate (Hernán et al., 2022). This includes defining eligibility criteria, treatment strategies, treatment assignment, start of follow-up, outcomes, causal contrast, and analysis plan. This framework is particularly useful because many common biases in pharmacoepidemiology arise from unclear alignment between treatment initiation, baseline covariate assessment, and start of follow-up. By aligning time zero with treatment initiation, target trial emulation can reduce immortal time bias, prevalent user bias, and ambiguity in causal interpretation.

Propensity score methods are commonly used to reduce measured confounding in observational drug studies. The propensity score represents the probability of receiving a treatment conditional on observed baseline covariates. It can be applied through matching, stratification, covariate adjustment, or inverse probability of treatment weighting. These approaches aim to improve balance between treatment groups on measured confounders and make comparison groups more similar. However, propensity score methods cannot address unmeasured confounding, poorly measured variables, or inappropriate adjustment for variables affected by treatment. Therefore, propensity score diagnostics, such as covariate balance assessment using standardized mean differences, should be reported rather than relying only on model fit or p-values.

In longitudinal pharmacoepidemiologic studies, time-varying confounding is a major challenge. This occurs when a variable changes over time, predicts future treatment, and is also affected by previous treatment. Standard regression adjustment may produce biased estimates in this situation because it may adjust away part of the treatment effect or fail to appropriately account for treatment-confounder feedback. MSMs, usually implemented using inverse probability weighting, are designed to address this problem by creating a weighted pseudo-population in which treatment is independent of measured time-varying confounders (Robins et al., 2000). Other g-methods, including the parametric g-formula and g-estimation, can also be used to estimate causal effects in the presence of time-varying exposures, treatment switching, censoring, and dynamic treatment strategies (Mansournia et al., 2017).

Instrumental variable approaches may be considered when unmeasured confounding is likely and a valid instrument is available. An instrumental variable should be associated with treatment selection, affect the outcome only through its effect on treatment, and not share common causes with the outcome. In pharmacoepidemiology, potential instruments may include physician prescribing preference, regional variation, formulary restrictions, or policy changes. However, these assumptions are strong and often difficult to verify, so instrumental variable analyses require careful justification and sensitivity analyses.

Sensitivity analyses are essential for assessing the robustness of causal interpretations. These may include alternative exposure definitions, different lag or induction periods, restrictions to new users, active-comparator analyses, negative control outcomes or exposures, quantitative bias analysis, assessment of unmeasured confounding, and complete-case *versus* imputed analyses for missing data. Rather than being optional, sensitivity analyses should be planned as part of the study design because they help determine whether findings are consistent across plausible assumptions.

Together, DAGs, target trial emulation, propensity score methods, g-methods, instrumental variable approaches, and sensitivity analyses provide a methodological framework for strengthening causal interpretation in pharmacoepidemiology. Importantly, these methods do not eliminate bias automatically. Their validity depends on the clarity of the research question, the quality of the data, correct temporal alignment of exposure and outcome, appropriate measurement of confounders, and transparent reporting of assumptions.

### 4.7 Biases and limitations

Biases are a major concern in pharmacoepidemiology, as they can distort associations between drug exposures and outcomes, leading to incorrect conclusions. These biases may arise at different stages of the research process, including study design, data collection, operationalization of variables, and analysis. Key categories include confounding, selection bias, information (measurement) bias, and time-related biases (Table 5). Recognizing, minimizing, and transparently reporting these biases is essential for producing valid and reliable real-world evidence.

**TABLE 5:** Common biases in pharmacoepidemiology and their impact.

Bias type	Description/Example	Impact on study results
Confounding by indication	Drug prescribed due to disease severity	Over/underestimation of effect
Immortal time bias	Misclassification of unexposed time as exposed	Exaggerated treatment benefit
Misclassification bias	Errors in exposure/outcome classification	Diluted or spurious associations
Healthy user bias	Healthier behaviors among adherent patients	Overestimation of benefit

#### 4.7.1 Confounding

Confounding occurs when the association between exposure and outcome is distorted by a third variable related to both.

##### 4.7.1.1 Confounding by indication

Occurs when the reason for prescribing a drug, such as disease severity, is itself associated with the outcome, making it difficult to distinguish the drug effect from the underlying disease effect (Prada-Ramallal et al., 2019; Acton et al., 2023). This can be reduced through careful comparator selection, new-user designs, active-comparator designs, measurement of disease severity where possible, propensity score methods, and sensitivity analyses (Prada-Ramallal et al., 2019; Acton et al., 2023).

##### 4.7.1.2 Unmeasured or residual confounding

Arises when relevant confounders are not captured or are inadequately measured in the data (Prada-Ramallal et al., 2019; Brown et al., 2024; Fu et al., 2021). This is common in routinely collected data, where lifestyle factors, disease severity, adherence, over-the-counter medication use, and socioeconomic factors may be incomplete. Researchers can partially address this through proxy variables, negative control analyses, instrumental variable methods when appropriate, and quantitative bias analysis (Prada-Ramallal et al., 2019; Brown et al., 2024; Fu et al., 2021).

##### 4.7.1.3 Healthy user/adherer bias

Occur when individuals who initiate or adhere to preventive therapies also engage in healthier behaviors, leading to overestimation of treatment benefits (Prada-Ramallal et al., 2019; Acton et al., 2023; Olawore et al., 2025). These biases may be reduced by selecting clinically comparable active comparators, adjusting for healthcare utilization and preventive care behaviors, and using negative control outcomes to detect residual healthy-user effects. (Prada-Ramallal et al., 2019; Acton et al., 2023; Olawore et al., 2025).

#### 4.7.2 Selection bias

Selection bias arises when the study population is not representative of the target population or when inclusion is related to exposure and outcome.

##### 4.7.2.1 Channeling bias (confounding by contraindication)

Occurs when certain treatments are preferentially prescribed to specific patient groups based on clinical characteristics, contraindications, perceived risk, or previous treatment history (Prada-Ramallal et al., 2019; Acton et al., 2023). This can be reduced by using active comparators with similar indications, restricting them to comparable clinical subgroups, and clearly describing the prescribing context (Prada-Ramallal et al., 2019; Acton et al., 2023).

##### 4.7.2.2 Loss to follow-up (informative censoring)

Occur when patients who leave the database, discontinue treatment, switch therapy, or are censored differ systematically from those who remain under observation (Prada-Ramallal et al., 2019; Fu et al., 2021). Inverse probability of censoring weights and sensitivity analyses can be used when censoring is related to patient characteristics or prognosis (Prada-Ramallal et al., 2019; Fu et al., 2021).

##### 4.7.2.3 Prevalent user bias

Occurs when long-term users are included in the exposed group. This may exclude early adverse events and select patients who have already tolerated or benefited from treatment, leading to biased estimates (Prada-Ramallal et al., 2019; Fu et al., 2021; Acton et al., 2023). A new-user design is generally preferred because it aligns baseline covariate assessment and start of follow-up with treatment initiation (Prada-Ramallal et al., 2019; Fu et al., 2021; Acton et al., 2023).

#### 4.7.3 Information (measurement) bias

Information bias refers to systematic errors in measuring exposure, outcome, or covariates.

##### 4.7.3.1 Misclassification bias

Occurs when exposure or outcome status is incorrectly classified, such as using prescription or dispensing records as a proxy for actual medication intake (Prada-Ramallal et al., 2019; Brown et al., 2024; Acton et al., 2023; Hall et al., 2020). Exposure misclassification may be reduced by clearly defining exposure windows, grace periods, dose changes, discontinuation, and switching. Outcome misclassification may be reduced by using validated diagnostic codes, laboratory results, procedure codes, or chart review when available (Prada-Ramallal et al., 2019; Brown et al., 2024; Acton et al., 2023; Hall et al., 2020).

##### 4.7.3.2 Missing Data

Occur when information on exposures, outcomes, covariates, or follow-up is incomplete (Prada-Ramallal et al., 2019; Fu et al., 2021; Acton et al., 2023). The impact of missing data depends on the missingness mechanism. Complete-case analysis may be appropriate in limited situations but can introduce bias if missingness is related to exposure or outcome. Multiple imputation, missingness indicators, inverse probability weighting, and sensitivity analyses may be considered depending on the extent and mechanism of missingness (Prada-Ramallal et al., 2019; Fu et al., 2021; Acton et al., 2023).

#### 4.7.4 Time-related bias

Time-related bias occurs when the timing of exposure, outcome, eligibility, or follow-up is incorrectly defined. These biases are especially important in pharmacoepidemiology because medication use changes over time.

##### 4.7.4.1 Immortal time bias

Occurs when a period during which the outcome cannot occur is incorrectly classified as exposed time, often leading to exaggerated treatment benefits (Suissa and Dell’Aniello, 2020; Suissa, 2008; Pazzagli et al., 2018). This can be prevented by aligning time zero with treatment initiation, using time-dependent exposure definitions when appropriate, and ensuring that eligibility, exposure classification, and follow-up begin at the same point (Suissa and Dell’Aniello, 2020; Suissa, 2008; Pazzagli et al., 2018).

##### 4.7.4.2 Time-lag, time-window, and immeasurable time biases

Arise from incorrect alignment of disease stage, exposure assessment period, follow-up duration, or data capture (Prada-Ramallal et al., 2019; Suissa and Dell’Aniello, 2020; Pazzagli et al., 2018; Oh et al., 2021). These biases can be reduced by selecting clinically comparable treatment groups, defining biologically plausible risk windows, applying appropriate lag periods, and accounting for hospitalization or other periods during which outpatient medication use may not be measurable (Prada-Ramallal et al., 2019; Suissa and Dell’Aniello, 2020; Pazzagli et al., 2018; Oh et al., 2021).

#### 4.7.5 Limitations of pharmacoepidemiology studies

Beyond methodological biases, pharmacoepidemiologic studies are subject to several system-level limitations, particularly when using routinely collected healthcare data. These include data quality limitations, such as coding errors, inconsistencies, and incomplete capture of clinical information (Prada-Ramallal et al., 2019), as well as a lack of clinical granularity, where important variables like disease severity, laboratory values, and lifestyle factors are often unavailable or poorly measured (Haque et al., 2024). Residual confounding may persist despite the use of advanced analytical methods due to unmeasured or inadequately captured variables. In addition, issues related to generalizability (external validity) may arise, as findings from specific populations or healthcare systems may not be applicable to other settings (Prada-Ramallal et al., 2019; Brown et al., 2024; Fu et al., 2021). Regulatory and ethical constraints, including data access restrictions, privacy regulations, and governance frameworks, can further limit data linkage and study design (Prada-Ramallal et al., 2019; Smit et al., 2023). Finally, while machine learning approaches are increasingly used, artificial intelligence limitations—particularly challenges related to model transparency, interpretability, and explainability—remain important considerations, especially in causal inference contexts (Stiglic et al., 2020). Recognizing both methodological biases and system-level limitations is essential for accurate interpretation and transparent reporting of pharmacoepidemiologic findings.

### 4.8 Reporting and interpretation

Transparent and standardized reporting is essential in pharmacoepidemiology to ensure reproducibility, validity, and comparability of research. Several guidelines and resources have been developed specifically for this field.

#### 4.8.1 STROBE (strengthening the reporting of observational studies in epidemiology)

Provides a general framework for reporting observational studies, including cohort, case-control, and cross-sectional designs. Researcher can use the STROBE checklist at https://www.strobe-statement.org/checklists/. (Elm et al., 2007).

#### 4.8.2 RECORD (reporting of studies conducted using observational routinely collected health data)

Extends STROBE for studies using routinely collected health data, such as electronic health records and claims databases (Benchimol et al., 2015). The checklist can be accessed at http://www.record-statement.org/checklist.php.

#### 4.8.3 RECORD-PE (RECORD for pharmacoepidemiology)

Further extends RECORD with items specific to pharmacoepidemiology, addressing complexities like exposure definitions, confounding, and data linkage (Langan et al., 2018). The RECORD-PE checklist and explanations are available http://www.record-statement.org/checklist-pe.php.

#### 4.8.4 HARPER (harmonized protocol template to enhance reproducibility)

Provides structured guidance for protocol development and reporting to improve transparency and reproducibility in pharmacoepidemiologic research (Wang et al., 2022).

#### 4.8.5 START-WE (structured template for assessment and reporting of pharmacoepidemiologic studies using real-world evidence)

Offers a structured framework for reporting studies using real-world data, with emphasis on methodological transparency and consistency (Wang et al., 2021).

#### 4.8.6 ISPE guidelines for good pharmacoepidemiology practice (GPP)

Comprehensive guidance on planning, conducting, and reporting pharmacoepidemiologic research, including protocol development, study conduct, communication, and adverse event reporting (ISPE, 2016).

#### 4.8.7 Additional resources: REPEAT initiative (real world evidence transparency initiative)

Provides tools and guidance to improve transparency, reproducibility, and validity in studies using real-world data, available at: https://www.repeatinitiative.org/.

## 5 Future directions in pharmacoepidemiology

Pharmacoepidemiology is rapidly evolving, driven by advances in data science, the integration of RWD with RCT evidence, international collaborations, and the growing influence of regulatory science.

### 5.1 AI and machine learning in drug safety

The explosion of healthcare data has created opportunities for artificial intelligence and machine learning to enhance drug safety surveillance. These technologies enable the analysis of large, complex datasets to detect rare adverse drug reactions, predict patient phenotypes, and improve outcome prediction. Machine learning models, including natural language processing, are increasingly used to identify drug-drug interactions and optimize medication use. In parallel, the integration of digital health technologies, such as wearable devices and mobile health applications, is enabling continuous data capture and supporting more real-time monitoring of medication safety and effectiveness. However, challenges remain regarding model interpretability, transparency, and clinical applicability, particularly in causal inference settings (Sabaté and Montané, 2023; Burden, 2019).

### 5.2 Real-world data integration with RCT evidence: opportunities, limitations and applicability

Pharmacoepidemiology is essential for generating real-world evidence (RWE) that complements randomized controlled trials (RCTs), particularly for populations underrepresented in trials, such as older adults, pregnant women, patients with multimorbidity, and those receiving multiple medications. The integration of real-world data (RWD), including electronic health records, claims data, disease registries, and pharmacovigilance databases, with RCT findings can provide a more comprehensive understanding of drug effectiveness and safety in routine clinical practice (Sabaté and Montané, 2023; Tanaka et al., 2015; Laroche et al., 2019). In this context, target trial emulation has emerged as a key methodological framework, enabling researchers to design observational studies that explicitly mimic randomized trials, improve causal interpretation, and reduce bias (Hernán et al., 2022). In addition, the adoption of common data models, such as the OMOP Observational Medical Outcomes Partnership model (Voss et al., 2015), facilitates standardized data structures and enables large-scale, reproducible analyses across multiple databases.

Despite these advantages, RWD has important limitations that require critical evaluation. Routinely collected healthcare data are primarily generated for clinical care, reimbursement, or administrative purposes rather than research, and therefore data quality may vary across healthcare systems, institutions, and databases (Zheng et al., 2025; Prada-Ramallal et al., 2019; Alarkawi et al., 2018). Key clinical variables, such as disease severity, laboratory values, lifestyle factors, medication adherence, socioeconomic indicators, and over-the-counter medication use, may be missing, incompletely captured, or inconsistently recorded (Zheng et al., 2025; Prada-Ramallal et al., 2019; Alarkawi et al., 2018). Missing data can introduce bias, particularly when missingness is related to exposure, outcome, or prognosis. Coding inaccuracies may also lead to exposure, outcome, or covariate misclassification, as diagnostic and procedure codes may not always reflect validated clinical events, and dispensing or prescription records may not accurately represent actual medication intake (Zheng et al., 2025; Prada-Ramallal et al., 2019; Alarkawi et al., 2018). These limitations highlight the need for validated definitions, sensitivity analyses, transparent reporting of missingness, and cautious interpretation of findings derived from RWD.

Ethical and governance considerations are also central to the use of RWD. Researchers must ensure appropriate protection of patient privacy, data security, consent procedures where applicable, and governance of data access and linkage. Large linked datasets may carry a risk of re-identification, particularly when rare diseases, small subgroups, or granular geographic information are included. Transparency in study protocols, analytical decisions, and reporting is therefore essential to maintain public trust and ensure responsible use of routinely collected health data. In addition, researchers should consider whether certain groups are underrepresented or poorly captured in available databases, as this may affect equity, generalizability, and the interpretation of real-world evidence.

The applicability of RWD-based pharmacoepidemiology also differs across settings. In low- and middle-income countries (LMICs), pharmacoepidemiologic research may be limited by fragmented healthcare data systems, incomplete electronic health records, limited linkage between prescribing, dispensing, laboratory, and outcome data, variable coding accuracy, and underdeveloped pharmacovigilance infrastructure (Kiguba et al., 2023; Mottla et al., 2023; Shafi et al., 2024; Hollingworth and Kairuz, 2021). Limited drug utilization data, low adverse-event reporting rates, and shortages of trained personnel may further restrict robust signal detection, active surveillance, and risk estimation (Kiguba et al., 2023; Mottla et al., 2023; Shafi et al., 2024; Hollingworth and Kairuz, 2021). In addition, contextual factors that strongly influence medicine use in LMICs, such as medication cost, access barriers, traditional medicine use, health literacy, and communication barriers, may be poorly captured in routinely collected data or in adherence tools developed in high-income settings (Kiguba et al., 2023; Hollingworth and Kairuz, 2021; Khoiry et al., 2023).

These challenges may affect exposure ascertainment, outcome validation, confounder measurement, follow-up completeness, and the generalizability of findings. However, LMIC settings also provide important opportunities for locally relevant pharmacoepidemiologic research, particularly through hospital databases, insurance claims, disease registries, electronic prescribing systems, drug utilization studies, and national pharmacovigilance programs. Broader applicability can be strengthened by standardizing coding practices, using internationally recognized drug classification systems such as WHO ATC/DDD methods, adopting common data models where feasible, validating key variables, improving governance frameworks, and building methodological capacity in pharmacoepidemiology and pharmacovigilance (Prada-Ramallal et al., 2019; Hollingworth and Kairuz, 2021; Man and Pottegård, 2025). Therefore, while RWD can strengthen evidence generation and complement RCTs, its interpretation requires careful attention to data quality, bias, ethics, and context-specific applicability.

### 5.3 International collaborations for multinational studies

Global collaboration is increasingly important in pharmacoepidemiology, enabling the pooling of data across diverse populations and healthcare systems. These efforts enhance the generalizability of findings, facilitate the study of rare outcomes, and support the development of harmonized data standards. Several large-scale initiatives exemplify this trend, including DARWIN EU (Data Analysis and Real-World Interrogation Network), OHDSI (Observational Health Data Sciences and Informatics), the Sentinel Initiative (U.S. FDA), and data standardization frameworks such as openEHR (Haber et al., 2025; Palomar-Cros et al., 2025). These initiatives support distributed data networks, standardized analytics, and large-scale evidence generation across countries and healthcare systems. Continued collaboration is essential to advance methodological innovation and improve global drug safety and effectiveness monitoring.

### 5.4 The role of regulatory science

Regulatory science is playing a larger role in shaping pharmacoepidemiology by guiding the use of RWE in drug approval, post-marketing surveillance, and risk management. Regulatory agencies are adopting new guidelines and frameworks to incorporate pharmacoepidemiologic evidence into decision-making, ensuring that drug safety and effectiveness are continuously evaluated throughout the product lifecycle (Sabaté and Montané, 2023; Tanaka et al., 2015). In this evolving landscape, approaches such as target trial emulation and advanced causal inference methods are becoming central to regulatory evaluation of real-world data, supporting more reliable and transparent evidence generation.

### 5.5 Real-time pharmacovigilance

Advances in data infrastructure and analytics are enabling a shift toward real-time pharmacovigilance, where adverse drug events can be detected, monitored, and evaluated more rapidly using continuously updated healthcare data. The integration of electronic health records, claims databases, and digital health technologies allows near real-time signal detection and risk assessment, improving the timeliness of safety evaluations (Lavertu et al., 2021). In addition, the use of distributed data networks and standardized data models supports scalable pharmacovigilance across multiple healthcare systems while maintaining data privacy. These developments are particularly relevant for post-marketing surveillance, where timely identification of safety signals is critical (Liu et al., 2019). Despite these advances, challenges remain regarding data quality, signal validation, and the balance between rapid detection and false-positive findings.

## 6 Conclusion

Pharmacoepidemiology stands at the intersection of pharmacology, epidemiology, and clinical practice, offering essential insights into the real-world use, safety, and effectiveness of medications. As this review has outlined, the field encompasses a broad range of themes—from pharmacovigilance and drug utilization research to comparative effectiveness and regulatory science—supported by a diverse array of data sources, study designs, and statistical tools. For early-career researchers and students, understanding foundational concepts such as exposure definitions, confounding, and study design selection is crucial for conducting robust pharmacoepidemiologic investigations. Emphasis must also be placed on recognizing biases and adhering to rigorous reporting standards like STROBE and RECORD. This review highlights these elements within a structured, workflow-oriented framework designed to support early-career researchers in navigating the main stages of pharmacoepidemiologic research. By following a structured workflow—from formulating a clear PICO question to selecting appropriate databases and statistical models—researchers can ensure scientific rigor and policy relevance. Ultimately, pharmacoepidemiology empowers evidence-based decision-making across clinical, public health, and regulatory domains, and serves as a critical bridge between scientific discovery and real-world patient care.
